# BH3 Profiling Identifies Apoptotic Priming by SREBP-1/-2 Inhibition and Synergistic Anti-Glioblastoma Activity When Combined with ABT-263 In Vitro

**DOI:** 10.3390/pharmaceutics18101327

**Published:** 2026-10-20

**Authors:** Davide Tosin, Felix Seyfried, Annika Hajosch, Dominik Schlotter, Letizia Santinelli, Mike-Andrew Westhoff, Markus Siegelin, Christian Rainer Wirtz, Georg Karpel-Massler

**Affiliations:** 1 Department of Neurosurgery, Ulm University Medical Center, 89081 Ulm, Germany; 2 Department of Pediatric Hematology, Oncology and Stem Cell Transplantation, University Medical Center Regensburg, 93053 Regensburg, Germany; 3 Institute of Neurogenetics, University of Lübeck, 23562 Lübeck, Germany; 4 Department of Pediatrics and Adolescent Medicine, Ulm University Medical Center, 89081 Ulm, Germany; 5 Department of Pathology and Cell Biology, Columbia University Medical Center, New York, NY 10032, USA

**Keywords:** glioblastoma, fatostatin, ABT-263, SREBP-1/2, Bcl-xL/Bcl-2, cancer metabolism

## Abstract

**Background/Objectives:** Glioblastoma exhibits profound therapy resistance driven in part by complex pro-survival signaling. We hypothesized that targeting lipid metabolism via SREBP-1/2 inhibition (fatostatin) might alter the apoptotic phenotype and facilitate an apoptotic response driven by BH3 mimetics (ABT-263) in glioblastoma in vitro. **Methods:** Dynamic BH3 profiling was performed to detect apoptotic priming. The combined inhibition of SREBP-1/2 and Bcl-xL/Bcl-2 was evaluated across 2- and 3-dimensional culture models. The cell lines used included established cell lines as well as primary cultures and glioma stem-like cells. Extracellular flux analysis was used to assess metabolic effects. Changes in migration were evaluated through time-lapse analysis, scratch, and Transwell assays. **Results:** BH3 profiling showed that treatment with fatostatin increased Bcl-xL/Bcl-2 reliance, while ABT-263 increased Mcl-1 dependence. The combination showed potent synergy in inhibiting viability and spheroid growth, driven at least in part by enhanced caspase-dependent apoptosis. Mechanistically, fatostatin induced dose-dependent Mcl-1 downregulation, sensitizing cells to ABT-263. Dual treatment suppressed oxidative phosphorylation and impaired cell migration, while exhibiting low toxicity in non-pathological cells. **Conclusions:** This study indicates that combined inhibition of SREBP-1/2 and Bcl-xL/Bcl-2 represents a promising therapeutic strategy for the treatment of glioblastoma. The efficacy of this dual approach potentially lies in the downregulation of Mcl-1 induced by fatostatin and suppression of oxidative phosphorylation. Further studies are warranted.

## 1. Introduction

Glioblastoma (GBM) represents the most common and aggressive primary brain malignancy in adults, characterized by diffuse invasiveness, high recurrence rates and frequent resistance to first-line therapy, including surgery, radiation, chemotherapy with temozolomide and tumor-treating fields, resulting in a poor prognosis and a median survival of less than 2 years [1]. The limited efficacy of current therapeutic regimens necessitates the identification of novel molecular targets and treatment strategies. Among the known hallmarks of GBM is metabolic dysregulation, specifically the upregulation of lipid metabolism [2,3]. GBM cells exhibit a high reliance on de novo lipogenesis for the rapid production of membrane components, energy storage and pro-survival signaling molecules which fuel aggressive proliferation [3,4]. Central to this metabolic reprogramming are the Sterol Regulatory Element-Binding Proteins (SREBPs), a family of transcription factors that control the expression of genes involved in lipogenesis and cholesterol uptake [3,5]. Specifically, SREBP-1 activation has been identified as a key metabolic vulnerability in GBM, promoting growth and survival [6]. SREBP signaling pathways were shown to be significantly upregulated in TMZ-resistant GBM cells in comparison to normal cells [2,3]. Consequently, pharmacological agents that interact with SREBPs represent a compelling therapeutic strategy. Fatostatin is an inhibitor of SREBP-1/2 presenting antineoplastic effects in vitro and in vivo across several cancer types [7,8]. With respect to GBM, in a recently published in vitro study, fatostatin was shown to inhibit TMZ-sensitive as well as TMZ-resistant cells [2]. The use of fatostatin thus offers a promising avenue for targeting an essential metabolic lifeline of GBM. Another known feature of GBM is its enhanced anti-apoptotic phenotype and particularly the overexpression of anti-apoptotic proteins belonging to the Bcl-2 family, such as Bcl-2 and Bcl-xL [9]. The role of Bcl-2 and Bcl-xL consists in preventing pro-apoptotic members of the Bcl-2 family, including BAX and BAK, from inducing cell death [10]. ABT-263 (navitoclax) is a potent BH3-mimetic that selectively inhibits Bcl-2 and Bcl-xL and hence promotes a pro-apoptotic shift in cellular homeostasis [11]. ABT-263 has already demonstrated efficacy in preclinical models and has advanced to clinical trials, particularly in hematologic malignancies, underscoring its translational potential [12,13]. GBM shows a remarkable intratumoral heterogeneity, which contributes to the development of resistance to therapies targeting a single cancer-promoting determinant [14]. Along this line, we hypothesized that disrupting the GBM cells’ lipid metabolism would create a critical vulnerability to be exploited in a multi-targeting approach. Dynamic BH3 profiling showed that treatment with fatostatin primes cells for apoptosis, creating a dependency on the anti-apoptotic Bcl-2 family proteins Bcl-xL and Bcl-2. The purpose of the present study was therefore to investigate whether interference with SREBP-1/2 via fatostatin empowers the anti-glioblastoma activity of Bcl-xL/Bcl-2 inhibition by ABT-263 in vitro.

## 2. Materials and Methods

### 2.1. Bioinformatics Analysis

Publicly available gene expression data and corresponding clinical metadata from the “Tumor Glioblastoma-TCGA-540-MAS5.0-u133a” dataset were analyzed using the R2: Genomics Analysis and Visualization Platform (Academic Medical Center, University of Amsterdam, The Netherlands, http://r2.amc.nl) with a focus on the sterol regulatory element-binding transcription factors (SREBF-1/2), responsible for encoding SREBP-1/-2. Overall survival associated with SREBF-1/2 expression levels was assessed using Kaplan–Meier analysis. The dataset was split into a high- and a low-expression cohort based on the median SREBF-1/2 expression values. Statistical significance was determined by the log-rank test.

### 2.2. Reagents

Fatostatin was acquired from Sigma Aldrich (St. Louis, MO, USA) and ABT-263 from Selleckchem (Houston, TX, USA). Stock solutions of fatostatin and ABT-263 were both prepared at concentrations of 10 mM in dimethyl sulfoxide (DMSO) and held in storage at a temperature of −20 °C. During all experiments, the ultimate concentration of DMSO remained below 0.1% (*v*/*v*).

### 2.3. Cell Cultures

The human GBM-derived cell line U251 was obtained from Sigma Aldrich (St. Louis, MO, USA) on 19 January 2017. Authenticity was confirmed via short tandem repeat profiling. U251 cells were passaged a maximum of 40 times and primary human GBM cells a maximum of 15 times prior to being re-thawed from their validated initial stocks. The primary human GBM cells ULM-GBM-PC38, ULM-GBM-PC40 and ULM-GBM-PC128 were derived from tumor resections performed at our institution and generated as detailed in previous work [15,16]. The primary neurosphere stem-like glioma cells, ULM-GBM-SC38 and ULM-GBM-SC40, were also sourced and characterized at our institution [17,18]. Human endothelial cells were kindly provided by Dr. Markus Hoenicka (Department of Cardiothoracic and Vascular Surgery, Ulm University Hospital, Ulm, Germany). U251 cells as well as primary human GBM cells were cultured in Dulbecco’s Modified Eagle Medium (DMEM) enriched with 10% Fetal Bovine Serum (FBS), 100 IU/mL penicillin, 100 μg/mL streptomycin, 4 mM glutamine and 1 mM sodium pyruvate (GIBCO, Invitrogen, Grand Island, NY, USA) as previously described [19,20]. ULM-GBM-PC128 were cultured in DMEM with the same aforementioned additions, except the overall FBS concentration was 20%. The primary neurosphere stem-like glioma cells were cultured in DMEM F12 (HAM) medium (GIBCO, Invitrogen, Grand Island, NY, USA), supplemented with human recombinant epidermal growth factor (Biomol GmbH, Hamburg, Germany), human recombinant basic fibroblast growth factor (Miltenyi Biotec, Bergisch Gladbach, Germany), and serum-free neuron culture supplement B27 (GIBCO, Invitrogen, Grand Island, NY, USA). All cells were incubated at 37 °C in a water-saturated atmosphere containing 5% CO_2_. Informed consent was secured from the patients or their next of kin, and all procedures adhered to the guidelines of the local ethics committee (approved ethics proposal No. 162/10, University of Ulm).

### 2.4. Dynamic BH3 Profiling

BH3 profiling was performed as described before [21]. Cells were exposed to fatostatin, ABT-263 or ABT-199 for 72 h. Afterward, they were permeabilized using digitonin and exposed to BH3 peptides. Cytochrome c release was analyzed by flow cytometry (Attune NxT Flow Cytometer, Thermo Fisher Scientific, Waltham, MA, USA). Cytochrome c median fluorescence intensities were determined and normalized to the median fluorescence intensities of cells treated with solvent as negative and Alamethicin as positive control.

### 2.5. Cell Viability Assays

GBM cells and primary neurosphere stem-like glioma cells were seeded at a density of 1500 cells/well in 96-well plates. Cellular viability was assessed using a 3-[4,5-dimethylthiazol-2-yl]-2,5-diphenyltetrazolium bromide (MTT)-based assay, performed as described previously [15]. Endothelial cells were seeded in plates coated with gelatine (Sigma Aldrich, St. Louis, MO, USA) containing endothelial cell growth medium MV (PromoCell, Heidelberg, Germany) and then incubated for 30 min at 37 °C before treatment.

### 2.6. Spheroid Assays

In order to test the therapeutic efficacy of fatostatin and ABT-263 within a three-dimensional (3D) culture model, cells were first seeded at a density of 0.35 × 10^5^ cells/well in 96-well plates, resuspended in a 20 μL matrix consisting of 80% Matrigel and 20% DMEM. This preparation was then incubated for 1 h at 37 °C. Subsequently, the cell/Matrigel construct was gently relocated to 12-well plates containing DMEM supplemented with 10% FBS. The spheroids were cultured for 7 days to allow growth, after which the medium was replaced with DMEM containing 1.5% FBS to initiate the experimental treatments. Cell viability and proliferation were quantified using the CellTiter-Glo^®^ Luminescent Cell Viability Assay (Version 2.0., Promega Corporation, Madison, WI, USA). Briefly, spheroids suspended in 100 μL of culture medium were transferred to 96-well plates. An equal volume (100 μL) of the CellTiter-Glo^®^ reagent was then added to each well, followed by a 10 min incubation at room temperature (RT) and transfer into opaque-wall plates to measure luminescence.

### 2.7. Assays for Apoptosis and Mitochondrial Membrane Potential

Apoptosis was detected by using either annexin V/propidium iodide (PI) or PI staining, followed by flow cytometry, as previously detailed [22,23]. In brief, 3 × 10^4^ cells were seeded in duplicate into 12-well plates and left to adhere overnight. Following the specified treatments, the cell supernatants and the enzymatically detached cells (using Trypsin/EDTA, Biochrom, Berlin, Germany) were harvested. Subsequently, the cells were centrifuged and rinsed twice with ice-cold Annexin V binding buffer (140 mM NaCl, 2.5 mM Ca^2+^, and 1 M 4-(2-hydroxyethyl)piperazine-1-ethanesulfonic acid (pH 7.4)). Cells were then resuspended in 100 µL of binding buffer and incubated with 2.5 µL of Annexin-V-FLUOS (Roche Diagnostics, Indianapolis, IN, USA) for 15 min at room temperature (RT). The cells were then washed once with ice-cold annexin V binding buffer before being resuspended in 300 µL of buffer. PI was then added to achieve a final concentration of 2.5 µM just before each measurement was taken. For each flow cytometric analysis, 10,000 events were recorded using a FACSCantoTM II flow cytometer (BD Biosciences, Franklin Lakes, NJ, USA). Quantitative analysis was performed using FlowJo software (version 7.6.5, Tree Star, Ashland, OR, USA). PI staining was performed as previously reported [22,23]. In short, cells were enzymatically detached and centrifuged for 5 min at 1800 rpm, then washed twice with PBS. Two hundred microliters of PI staining solution, consisting of 0.05% trisodium citrate dihydrate (Carl Roth, Karlsruhe, Germany), 0.05% triton-X100, and 0.05 mg/mL PI (Sigma Aldrich, St. Louis, MO, USA), was then added, and the cells were incubated for 30 min at 4 °C before flow cytometric analysis. To identify intrinsic apoptosis, staining with tetramethylrhodamine ethyl ester (TMRE) was conducted. Cell culture plates were centrifuged at 1300 rpm for 5 min before the supernatant was aspirated. A 200 nM TMRE (Thermo Fisher Scientific, Eugene, OR, USA) staining solution was then added, and the cells were incubated for 20 min at 37 °C. After this, the cells were enzymatically detached and centrifuged at 1800 rpm for 5 min, followed by the removal of the supernatant. The cells were then resuspended in 300 µL of PBS for subsequent flow cytometric analysis. The resulting data were analyzed using FlowJo software (version 7.6.5, Tree Star, Ashland, OR, USA).

### 2.8. Western Blot Analysis

Protein expression was evaluated utilizing Western blot analysis, as previously described [24], employing the following primary antibodies: anti-Mcl-1 (1:1000; #5453S, clone: D35A5, CST: Cell Signaling Technology, Danvers, MA, USA), anti-Bcl-2 (1:1000; #551098, BD Biosciences, Franklin Lakes, NJ, USA), anti-Bcl-xL (1:500; sc-1041, Santa Cruz Biotechnology, Inc., Santa Cruz, CA, USA or 1:500; #2764, clone 54H6, CST), anti-β-actin (1:2000, clone AC15; Sigma Aldrich, St. Louis, MO, USA), anti-Noxa (1:1000; #556585, clone 114C307.1, Enzo Life Sciences, Farmingdale, NY, USA), anti-human caspase-3 (1:1000; #9662, CST) and anti-human caspase-9 (1:1000; #9508 T, clone C9, CST). The secondary HRP-linked antibodies were also procured from CST (#7076S, #7074S).

### 2.9. Extracellular Flux Analysis

A total of 1 × 10^5^ cells were plated per well in XF96 V3 PS cell culture microplates (Agilent Technologies, Wilmington, DE, USA). Following a 24 h attachment period, cells underwent the specified treatments for an additional 24 h. The cells were then washed in XF assay medium containing 5 mM glucose (pH 7.5). The Mitochondrial Stress Test Kit (Agilent Technologies, Wilmington, DE, USA) was subsequently utilized according to the manufacturer’s protocol, involving the sequential injection of modulators: oligomycin (2 μM final concentration), FCCP (2 μM final concentration), and rotenone/antimycin A (0.5 μM final concentration). After that, analyses were performed with an Agilent Seahorse XFe96 (Agilent Technologies, Santa Clara, CA, USA).

### 2.10. Cell Migration and Motility Assays

Transmembrane migration (Transwell^®^, Corning, Corning, NY, USA) and scratch wound healing assays were used to evaluate cell migration. Time-lapse live cell imaging was employed to assess cell motility. For the Transwell^®^ assays, 3 × 10^4^ cells were seeded onto Transwell^®^ membranes (Corning, Corning, NY, USA) with an 8 µM pore size in DMEM containing 1.5% FBS. They were then allowed to migrate through the membranes toward a medium enriched with 10% FBS. These experiments followed the manufacturer’s recommendations. After 24 h, the top side of the membrane was wiped and washed three times with phosphate-buffered saline (PBS). The cells on the bottom side of the membrane were then fixed with methanol and stained with 4′6-diamidino-2-phenylindole (DAPI) before being mounted. Migrated cells were then determined by counting one high-power field at 10× magnification, in triplicate, for each treatment condition. Scratch wound healing assays were performed as described before [15]. Briefly, a sub-confluent layer of cells in 96-well plates was scratched with a 200 µL pipette tip. Sequential microscopic images were then taken at specific time points at 10× magnification, and the areas of the scratches were analyzed using the Incucyte^®^ software (v2021, Sartorius, Göttingen, Germany). To obtain time-lapse live cell imaging, 4 × 10^4^ cells/well were first seeded into 12-well plates. Microscopic images at 10× magnification were then captured using a live-imaging inverted video microscope (Zeiss Observer.Z1, Göttingen, Germany) every 30 min for a total duration of 24 h. Throughout this period, the cells were maintained under standard culture conditions (37 °C, 5% CO_2_, humidified atmosphere). Single-cell tracking was conducted using the MtrackJ plugin for ImageJ software (Version 1.53k, NIH, Washington, D.C., USA) [15]. Normalized “wind-rose” plots were generated using the chemotaxis and migration tool from Integrated BioDiagnostics (Ibidi, Martinsried, Germany).

### 2.11. Statistical Analysis

Statistical analysis was performed utilizing Prism (version 5.04, GraphPad, La Jolla, CA, USA). For comparisons between independent groups, statistical significance was determined using the Mann–Whitney U test in the case of 2 groups and ordinary one-way ANOVA followed by Tukey’s multiple comparison test to compare 3 or more groups. Dependent groups were analyzed with repeated measures ANOVA and Tukey’s multiple comparison test. A *p*-value of less than 0.05 (*p* < 0.05) was considered statistically significant. Combination indices and isobolograms were computed with the CompuSyn software (version 1.0.1, ComboSyn,., Paramus, NJ, USA).

## 3. Results

### 3.1. SREBF-1 Expression Correlates with Poor Prognosis in GBM Patients

**Figure 1:**
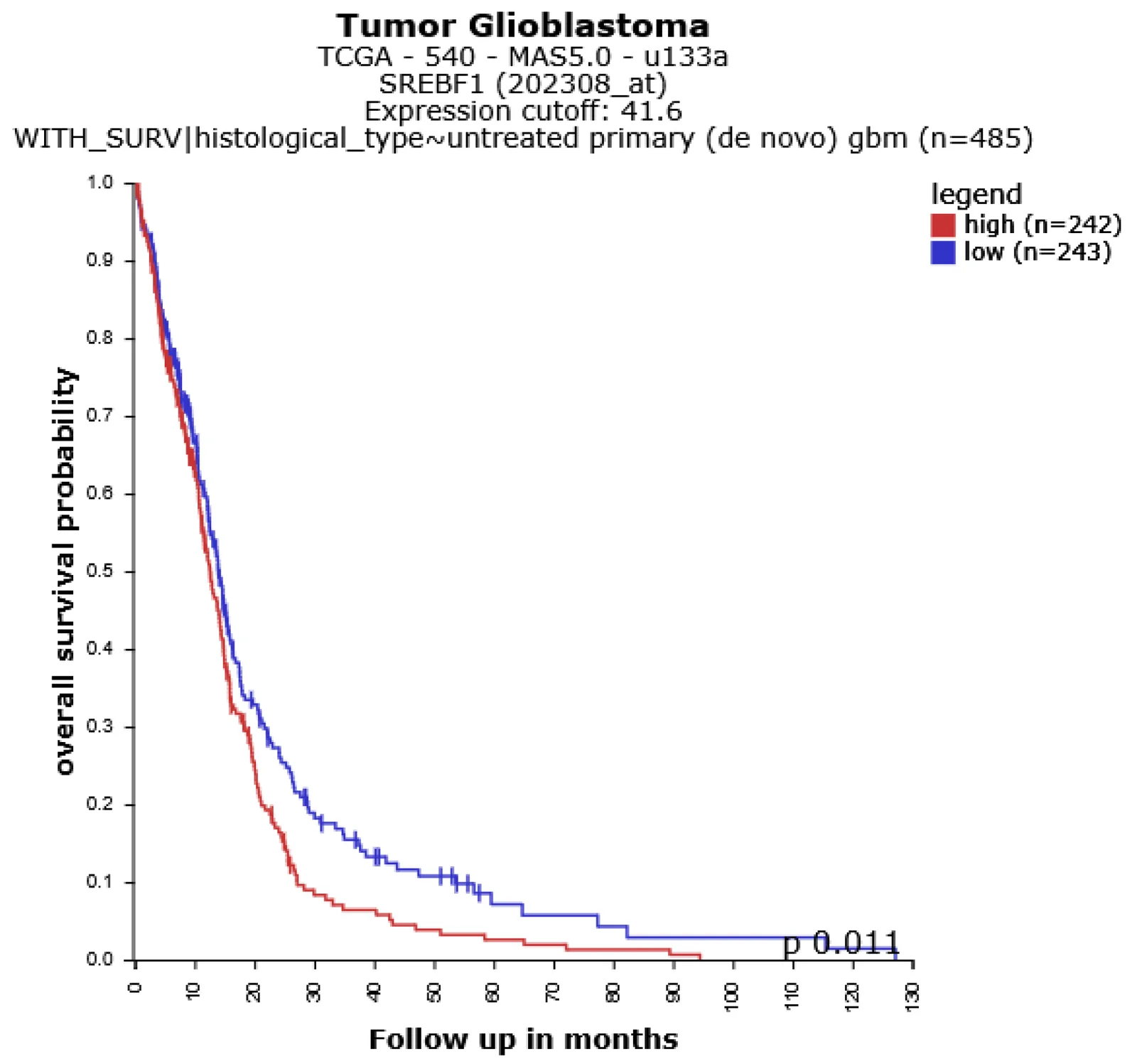
High SREBF-1 expression is associated with reduced survival in GBM. Kaplan–Meier analysis of overall survival in 485 patients with untreated primary GBM from the TCGA-540 dataset. Patients were stratified into high and low SREBF-1 expression groups based on the median expression cutoff. Statistical significance was determined using the log-rank test. The graph was generated using the R2 Genomics Analysis and Visualization Platform (Academic Medical Center, University of Amsterdam, The Netherlands, http://r2.amc.nl).

We first examined how the expression of SREBPs is associated with clinical outcomes in patients. Using the TCGA-540 dataset (*n* = 485), we observed that patients with high SREBF-1 expression levels exhibited significantly shorter overall survival compared to those with low expression (*p* = 0.011, Log-rank test; Figure 1). These findings highlight SREBP-1 as a potential therapeutic target and biomarker for poor prognosis in GBM.

### 3.2. Fatostatin Leads to Increased Dependence on Bcl-xL and Bcl-2

**Figure 2:**
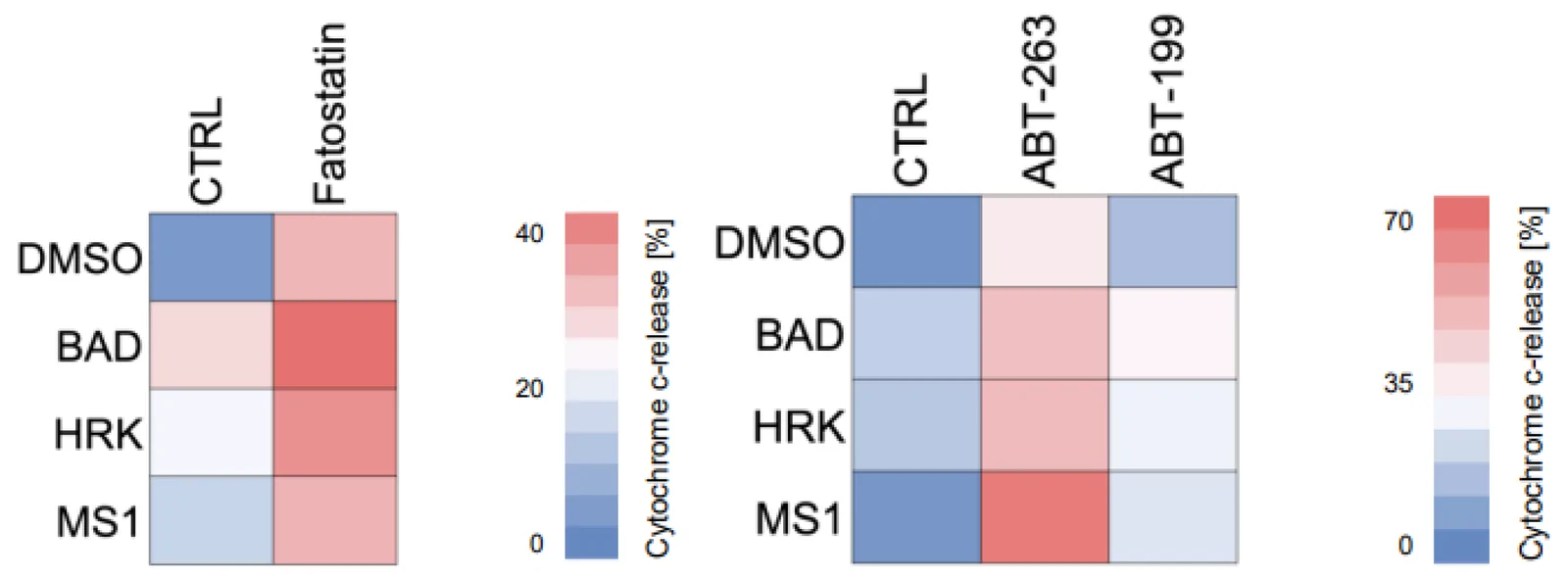
Treatment with fatostatin leads to increased dependence on Bcl-xL and Bcl-2, while treatment with ABT-263 leads to increased Mcl-1 dependence. U251 cells were treated for 72 h with 6.25 µM fatostatin, 0.5 µM ABT-263 or 0.5 µM ABT-199. Then, cells were harvested, permeabilized and subjected to treatment with the pro-apoptotic peptides BAD, HRK or MS1. Cytochrome c release was determined by flow cytometry. BAD inhibits BCL-2, HRK inhibits BCL-xL and MS1 inhibits Mcl-1.

We performed BH3-profiling to examine whether treatment with fatostatin affects dependencies on regulatory molecules of the intrinsic pathway of apoptosis. Treatment with fatostatin alone was related to a high cytochrome c release in U251 cells treated with BAD, a peptide that binds the anti-apoptotic proteins Bcl-2 and Bcl-xL (Figure 2). In contrast, U251 cells treated with the combined Bcl-xL/Bcl-2 inhibitor, ABT-263, presented a particularly high cytochrome c release when additionally subjected to the MS1 peptide, which binds the pro-survival protein Mcl-1. In other words, while fatostatin leads to a predominant dependence on the anti-apoptotic Bcl-2 family members Bcl-xL and Bcl-2, ABT-263 leads to increased dependence on Mcl-1 in GBM cells. These results suggest that dual treatment with fatostatin and ABT-263 might be a promising therapeutic approach. On the contrary, treatment of U251 cells with ABT-199 led to a rather low cytochrome c release concomitant with administration of either BAD, HRK or MS1 peptides.

### 3.3. Fatostatin Combined with ABT-263 Synergistically Reduces Cell Viability

**Figure 3:**
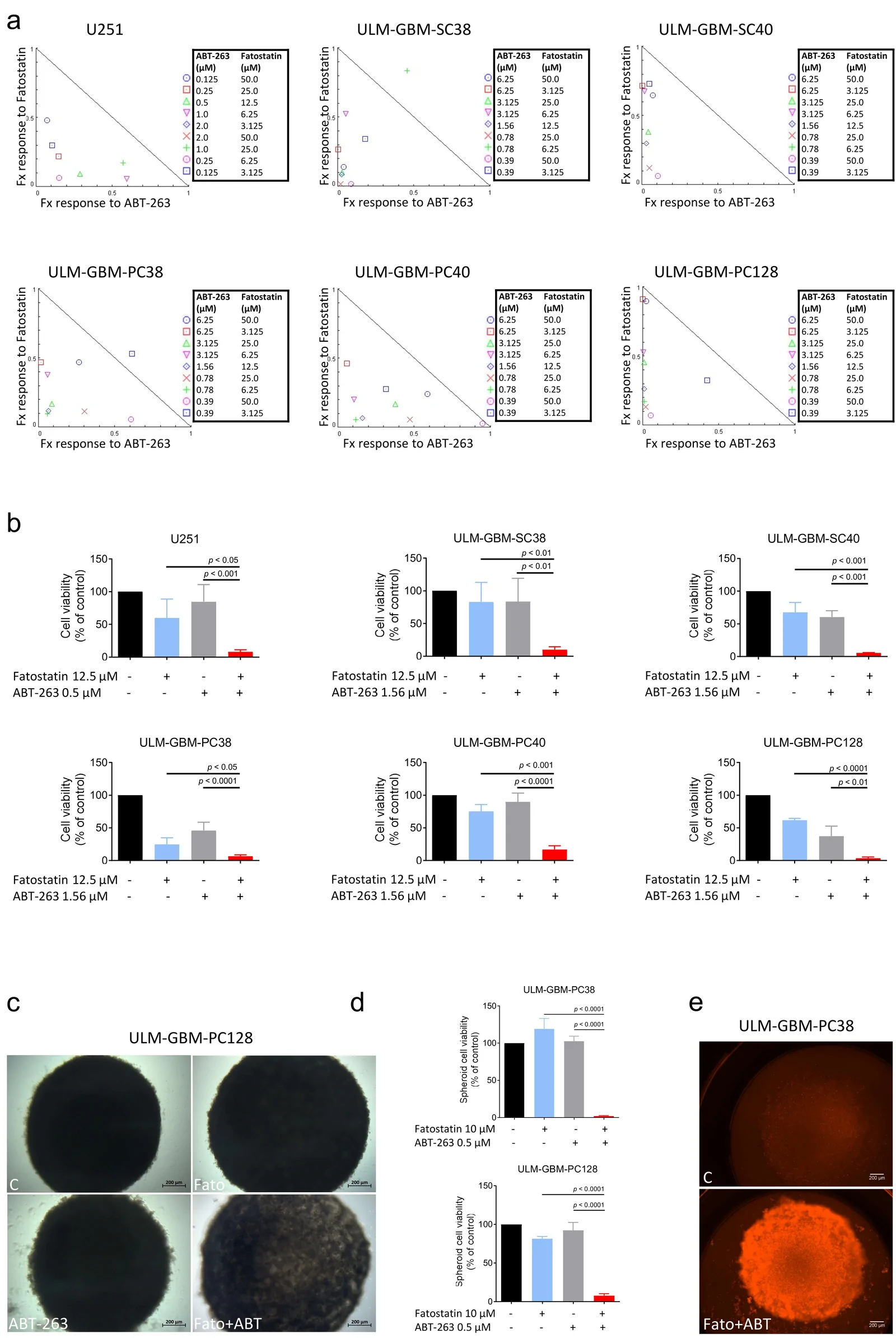
Combined treatment with fatostatin and ABT-263 synergistically reduces the viability of GBM cells; (**a**) U251 as well as primary-cultured GBM cells ULM-GBM-PC38, ULM-GBM-PC40, and ULM-GBM-PC128 and stem-like glioma cells ULM-GBM-SC38 and ULM-GBM-SC40 were treated for 72 h as indicated and then examined by MTT assay. Normalized isobolograms were computed using the CompuSyn software. While the connecting line represents additivity, data points below the diagonal line indicate a synergistic and those above the line an antagonistic drug–drug interaction. Data are representative of three independent experiments; (**b**) MTT assays were performed in U251 cells, primary-cultured GBM cells and stem-like glioma cells after treatment as outlined for (**a**). Columns, mean; bars, SD; *n* = 3; (**c**) Representative microphotographs of ULM-GBM-PC128 spheroids that were grown for 14 d and treated as indicated on d7, d9 and d12. Magnification: ×4, scale bar: 200 µm; (**d**) ULM-GBM-PC38 spheroids and ULM-GBM-PC128 spheroids were treated as described for (**c**) before quantifying cell viability using CellTiter-Glo^®^ assays. Columns, mean; bars, SD; *n* = 4; (**e**) Representative microphotographs of ULM-GBM-PC38 spheroids treated as indicated and stained with PI. Images were acquired using the EVOS M7000 (Thermo Fisher Scientific, Bothel, WA, USA) at 4× magnification, scale bar: 200 µm.

Based on the results of the BH3 profiling, we then used MTT assays and successively computed isobolograms (Figure 3a,b) as well as combination index (CI) diagrams (Figure S1a) to assess whether fatostatin and ABT-263 synergistically reduce the viability of GBM cells. In U251 cells as well as in primary cultures and glioma stem-like cells, we observed a synergistic reduction in cellular viability after combined treatment with fatostatin and ABT-263.

### 3.4. Dual Treatment with Fatostatin and ABT-263 Synergistically Inhibits the Growth of Spheroids

In order to further examine the combined treatment with fatostatin and ABT-263 in a three-dimensional setting, we grew spheroids of U251, ULM-GBM-PC38 and ULM-GBM-PC128 cells. As detected by microscopic imaging and subsequent luminescent cell viability assay (Figure 3c,d), dual treatment with fatostatin and ABT-263 reduced cellular density and impaired cellular cohesion within the spheroids when compared to treatment with either agent alone. Overall spheroid size after therapy did not change significantly compared to controls and single-agent treatments. In line with this reduction in cell density, intracellular ATP levels significantly decreased following the combination treatment (Figure 3d).

### 3.5. Dual Treatment with Fatostatin and ABT-263 Leads to Enhanced Apoptosis

**Figure 4:**
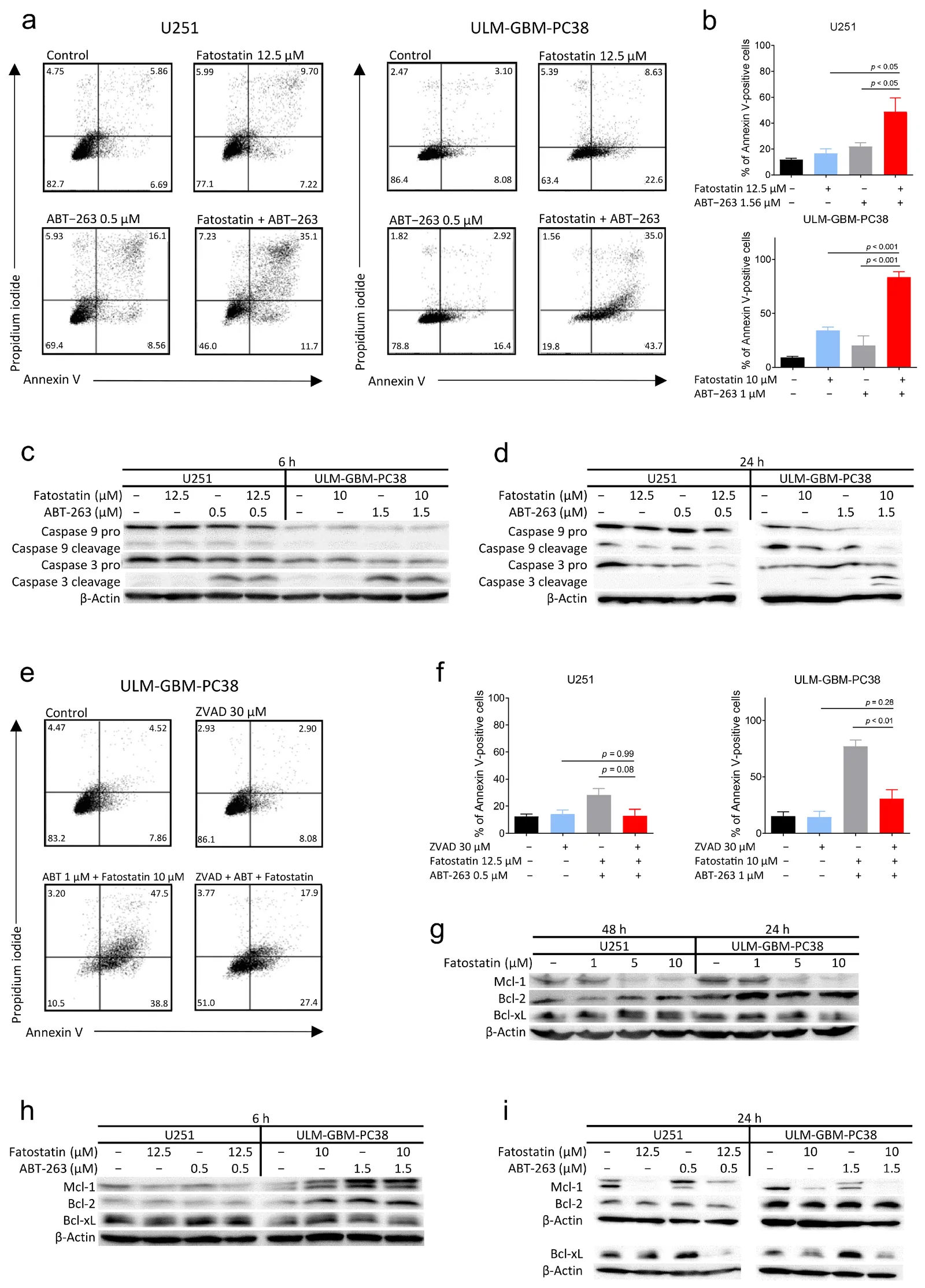
Combined treatment with fatostatin and ABT-263 induces activation of caspases and results in a pro-apoptotic shift in GBM cells. (**a**) Representative flow plots of U251 and ULM-GBM-PC38 cells, treated for 48 h and 24 h, respectively, as indicated. Cells were stained with annexin V/propidium iodide and underwent flow cytometric analysis. Data are representative of three independent experiments with a minimum of 10,000 events each. Matrix spillover coefficients applied to U251 cells: 13.9% for PerCP subtracted from the FITC channel, 0.1% for FITC subtracted from the PerCP channel. Matrix spillover coefficients applied to ULM-GBM-PC38: 7.0% for PerCP subtracted from the FITC channel, 0.1% for FITC subtracted from the PerCP channel; (**b**) quantitative representation of U251 and ULM-GBM-PC38 cells after treatment as outlined in (**a**). Columns, mean; bars, SD; *n* = 3; (**c**,**d**) After treating U251 and ULM-GBM-PC38 cells for 6 h (**c**) and 24 h (**d**) as indicated, whole-cell extracts were collected and subjected to Western blot analysis for caspase 3, cleaved caspase 3, caspase 9 and cleaved caspase 9 expression. ꞵ-Actin was employed as a loading control. Densitometric analysis was performed using Bio-1D^®^ (version 18.16g, Vilber, Eberhardzell, Germany); (**e**) representative flow plots of ULM-GBM-PC38 cells. After treating U251 cells for 48 h and ULM-GBM-PC38 for 24 h as indicated, cells were stained with annexin V/propidium iodide and analyzed by flow cytometry. Data are representative of five independent experiments with a minimum of 10,000 events each. Matrix spillover coefficients applied to ULM-GBM-PC38: 11.1% for PerCP subtracted from the FITC channel, 0.1% for FITC subtracted from the PerCP channel; (**f**) quantitative representation of U251 and ULM-GBM-PC38 cells after treatment as described in (**e**). Columns, mean; bars, SD; *n* = 5; (**g**–**i**) U251 and ULM-GBM-PC38 cells were first treated with fatostatin for 24 h (**g**) or fatostatin, ABT-263 and the combination for 6 h (**h**) or 24 h (**i**), as indicated. Whole-cell lysates were then collected and analyzed by Western blot for expression of Bcl-2, Bcl-xL and Mcl-1. Loading was controlled by β-Actin. Bio-1D^®^ (version 18.16g, Vilber, Eberhardzell, Germany) was used for densitometric analysis.

Staining with PI was strongly enhanced following dual treatment (Figure 3e). To investigate whether the inhibitory effect of the combination treatment is at least in part due to enhanced apoptosis, U251 and ULM-GBM-PC38 cells were analyzed by flow cytometry after staining with annexin V/PI. As depicted in Figure 4a,b, dual treatment was associated with a significant increase in the fraction of annexin V-positive (apoptotic) cells in comparison to either of the two monotherapies alone.

### 3.6. Combined Treatment with Fatostatin and ABT-263 Induces Activation of Caspases

In order to verify whether the observed pro-apoptotic response after dual treatment was dependent on caspase cleavage at the molecular level, we performed Western blot analyses. In both U251 and ULM-GBM-PC38 cells, fatostatin combined with ABT-263 was related to enhanced cleavage of caspase-3 both after 6 and 24 h of treatment (Figure 4a–d). In addition, annexin V/PI staining and flow-cytometric analysis were performed in U251 and ULM-GBM-PC38 cells in the absence or presence of the pan-caspase inhibitor zVAD-fmk in addition to the combined treatment. As shown in Figure 4e,f, zVAD-fmk attenuated the pro-apoptotic response mediated by the combination treatment.

### 3.7. Treatment with Fatostatin Results in a Dose-Dependent Downregulation of Mcl-1

To further investigate the effect of fatostatin at the molecular level, we analyzed U251 and ULM-GBM-PC38 cells focusing on the anti-apoptotic proteins of the Bcl-2 family Mcl-1, Bcl-2 and Bcl-xL. Treatment with fatostatin was found to decrease expression of Mcl-1 in a dose-dependent fashion (Figure 4g and Figure S2a). On the contrary, protein levels of Bcl-2 were increased after fatostatin monotherapy. In addition, when combined with ABT-263, fatostatin was observed to counteract ABT-263-mediated upregulation of Mcl-1, especially after treating both examined cell lines for 24 h (Figure 4h,i and Figure S2b,c). Moreover, in ULM-GBM-PC38, a downregulation of Bcl-2 and Bcl-xL was evidenced after 24 h of treatment with fatostatin as well as with both substances combined (Figure S2c).

### 3.8. Combined Treatment with Fatostatin and ABT-263 Suppresses OXPHOS in GBM Cells

**Figure 5:**
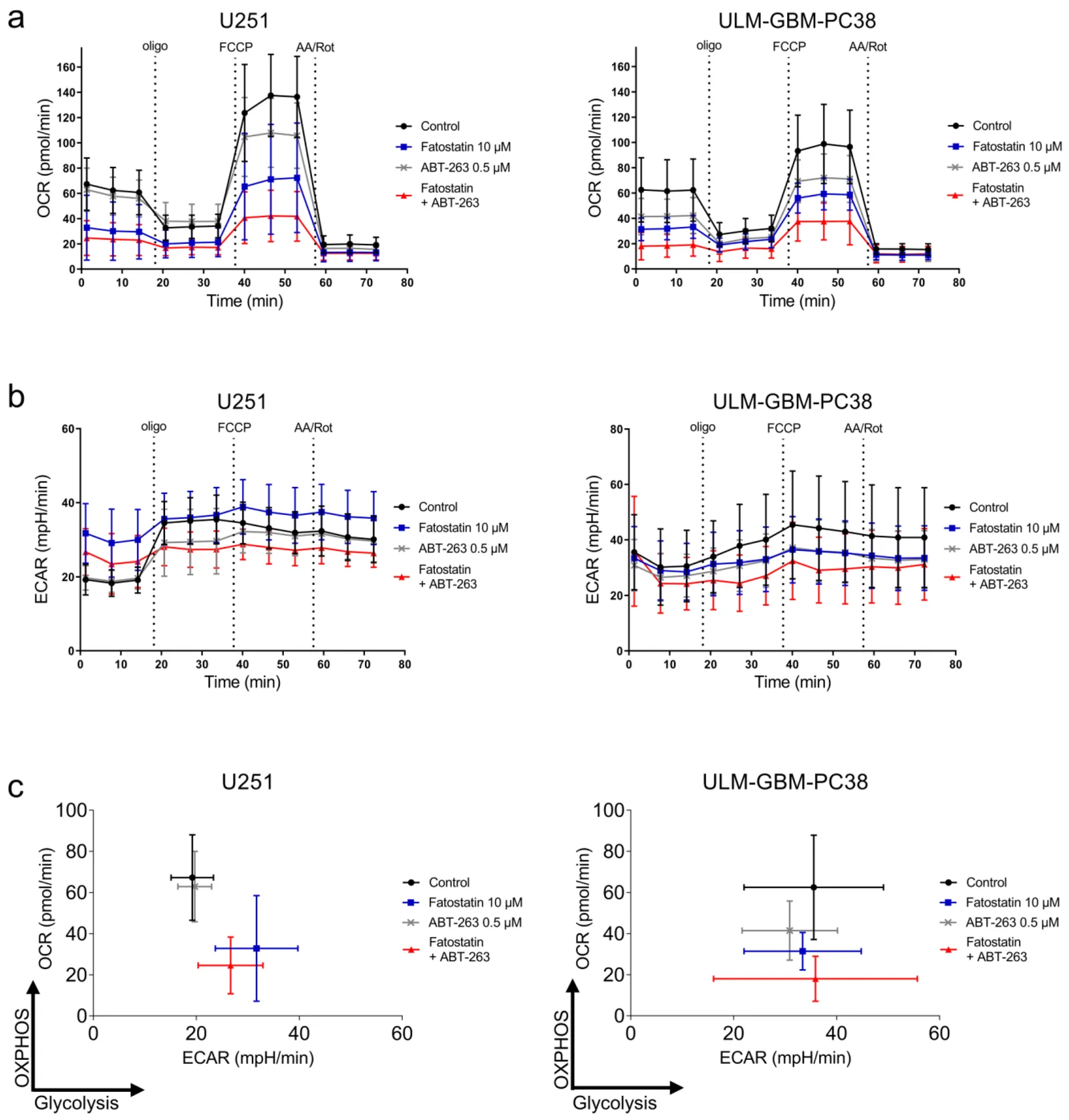
Fatostatin in combination with ABT-263 suppresses OXPHOS in GBM cells; (**a**) mitochondrial stress tests were performed in U251 and ULM-GBM-PC38 cells treated for 24 h as outlined. Oligomycin (oligo), FCCP and rotenone/antimycin A (AA/Rot) were sequentially injected, whilst extracellular flux analysis recording oxygen consumption rates (OCR) was performed. Data represent the means and SD, *n* = 3; (**b**) Extracellular acidification rates (ECAR) for U251 and ULM-GBM-PC38 cells treated as previously described in (**a**); (**c**) Graphical representation of baseline OCR/ECAR values. Data are presented as mean and SD; *n* = 3.

Based on the observation that the combination treatment leads to a significant decrease in ATP levels in spheroids (Figure 3d), we assessed whether the energy metabolism of GBM cells is reprogrammed by fatostatin and ABT-263. To this purpose, we used extracellular flux analyses and measured oxygen consumption rates (OCR) and extracellular acidification rates (ECARs). In both U251 and ULM-GBM-PC38 cells, dual treatment led to a reduction in OCR at baseline compared to either fatostatin or ABT-263 alone (Figure 5a–c). In U251 cells, ECARs at baseline were higher after dual treatment in comparison to single treatments, without this being observed in ULM-GBM-PC38 cells (Figure 5b,c). Besides, over the course of mitochondrial stress tests, OCR and ECAR remained suppressed in both cell lines subjected to the combination therapy compared to controls and to monotherapies (Figure 5a,b).

### 3.9. The Dual Treatment Shows Low Toxicity in Human Endothelial Cells

**Figure 6:**
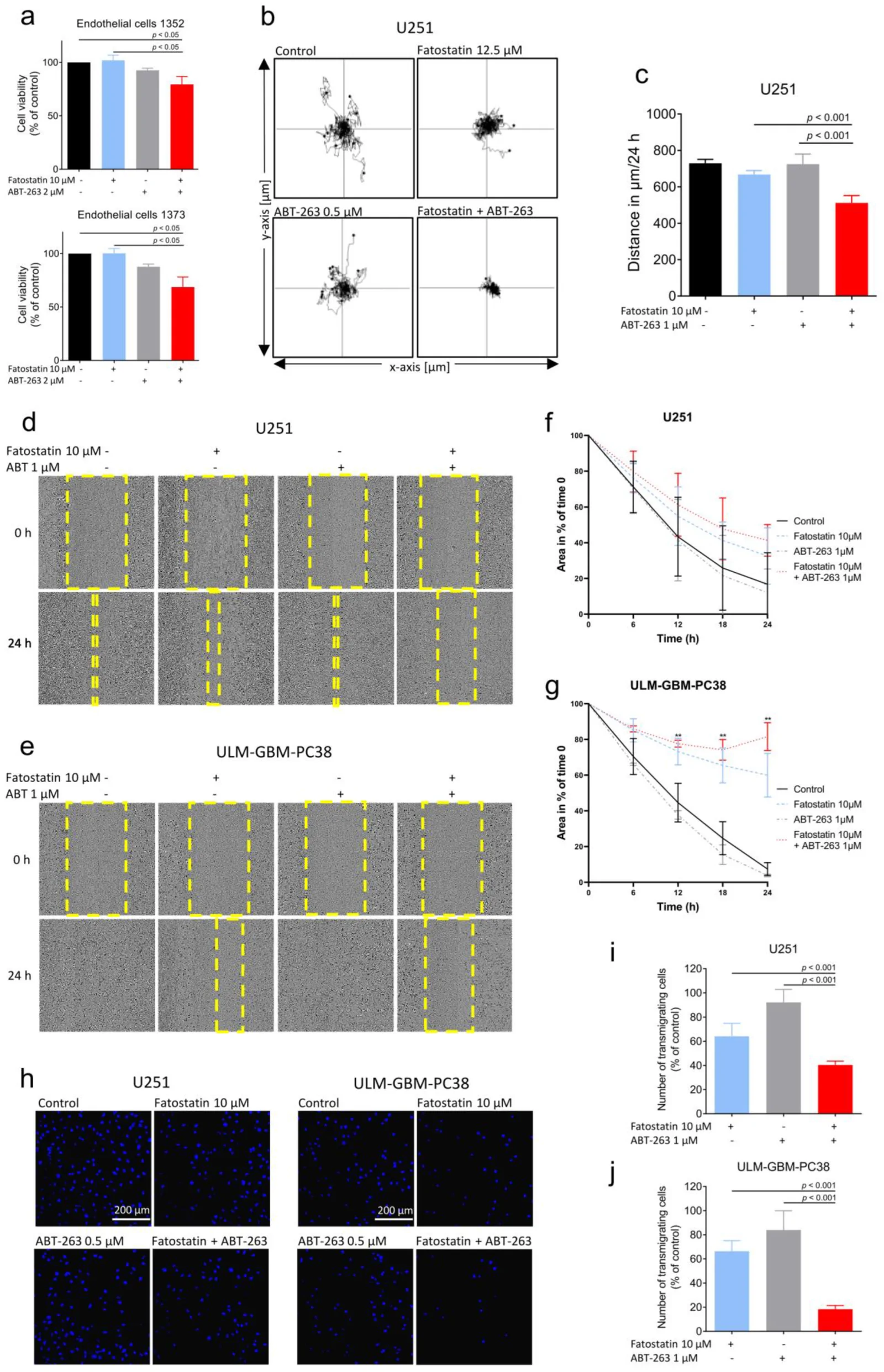
Treatment of GBM cells with fatostatin and ABT-263 combined results in enhanced anti-migratory activity; (**a**) human endothelial cells were analyzed by MTT assay 72 h after treatment with fatostatin and ABT-263 as indicated. Columns, mean; bars, SD; *n* = 3; (**b**) Representative wind-rose plots displaying the paths of 15 single cells per treatment condition during an observation period of 24 h. U251 cells were first seeded in 96-well plates and then underwent sequential microscopic imaging (magnification: 10×) over 24 h. Single-cell tracking was performed using the MtrackJ plugin for ImageJ software (Version 1.53k, NIH, Washington, D.C., USA). The tracks were aligned to start from the same initial position to facilitate comparison; (**c**) total distance covered by 45 cells within 24 h per treatment condition. Columns, mean; bars, SD; *n* = 3; (**d**,**e**) monolayers of sub-confluent U251 (**d**) and ULM-GBM-PC38 (**e**) cells were scratched prior to treatment with either solvent or fatostatin (10 μM)/ABT-263 (1 μM) following microscopic imaging at time 0 h and 24 h after infliction of the scratch (magnification: 10×) and analysis using the Incucyte^®^ software. Scale bar: 400 µm; (**f**,**g**) graphical representation of the quantitative assessment of scratch closure of cells at 6 h, 12 h, 18 h and 24 h after treatment as outlined in (**d**,**e**) compared to time 0 h. Data are presented as mean and SD; *n* = 3. ** signifies *p* < 0.01; (**h**) Representative microphotographs of U251 and ULM-GBM-PC38 cells (magnification: 10×). A total of 3 × 10^4^ cells were seeded onto a Transwell^®^ membrane and treated as indicated. Medium containing 10% FBS served as a chemoattractant. After 24 h, cells on the upper side of the membrane were wiped off, and those on the lower side were stained with DAPI prior to mounting; (**i**,**j**) quantitative representation of transmigrating U251 (**i**) and ULM-GBM-PC38 (**j**) cells treated as described in (**h**). One representative high-power field (hpf) per transwell^®^ membrane was counted, yieding a total of 3 hpf per treatment condition. Columns, mean; bars, SD; *n* = 3.

To investigate how fatostatin and ABT-263 combined affect non-pathological human cells, we applied the same therapy to human endothelial cells. Dual treatment significantly impaired the viability of human endothelial cells compared to control and to fatostatin alone (Figure 6a). Nevertheless, this effect was of a noticeably lesser degree than in GBM cells.

### 3.10. Fatostatin Combined with ABT-263 Inhibits the Migration of GBM Cells

Since GBM cells typically display dysregulated migratory activity, we then examined the random movement of U251 and ULM-GBM-PC38 cells after dual treatment. Cells were hence subjected to time-lapse analyses and as depicted in Figure 6b,c, fatostatin together with ABT-263 significantly reduced the migratory activity in comparison to the slight reduction observed after monotherapy with either agent. In addition, scratch assays were performed to investigate whether the dual treatment would also impair directed cell movement (Figure 6d–g). Fatostatin and ABT-263 combined led to a significantly reduced migration of cells into the scratch in ULM-GBM-PC38 cells, while this effect was less evident in U251 cells. In addition, we analyzed the cells’ ability to cross a collagen-coated porous membrane along a chemoattractant (FBS) gradient. As shown in Figure 6h–j, transmigration of U251 and ULM-GBM-PC38 cells was significantly reduced after the combination treatment compared to treatment with either agent alone. These results altogether indicate that fatostatin in combination with ABT-263 results in enhanced anti-migratory activity of GBM cells.

## 4. Discussion

GBM is characterized by an exceptionally poor prognosis, primarily stemming from its aggressive biological behavior and inherent resistance to conventional therapies. Given the vast intratumoral heterogeneity and therapy-induced plasticity of GBM, we posited that therapeutic strategies concurrently targeting multiple essential pathways would yield superior efficacy compared to approaches focused on a single molecular determinant. In this study, we explored a novel combination therapy designed to disrupt two key pillars of GBM cell survival: lipid metabolism and mitochondrially-mediated apoptosis. We selected fatostatin, an inhibitor of SREBP-1/2, and ABT-263, a BH3-mimetic targeting the anti-apoptotic proteins Bcl-xL and Bcl-2. The selection of these two agents was rationally supported by the following factors. First, our preliminary analysis of a large-scale transcriptomic dataset from TCGA highlighted that elevated expression of SREBF-1 was significantly correlated with shortened overall survival in patients with untreated de novo GBM. This clinical association suggests that SREBP-1 may serve as a critical driver of tumor progression and provides a robust rationale for targeting its pathway using inhibitors such as fatostatin. Second, BH3-profiling of an established GBM cell line revealed distinct dependencies induced by each monotherapy: ABT-263 treatment resulted in a heightened reliance on Mcl-1 for survival and, conversely, treatment with fatostatin alone increased cellular dependence on Bcl-xL/Bcl-2. This finding is critical because, while ABT-263 effectively neutralizes Bcl-xL/Bcl-2, its efficacy in GBM is known to be often limited by the compensatory overexpression of Mcl-1 [25,26]. Furthermore, Bcl-xL and Mcl-1 were shown to be the most overexpressed anti-apoptotic members of the Bcl-2 family in a recent investigation on patient-derived glioma stem-like cells [27]. The reciprocal dependencies observed strongly suggested that combining these two agents could overcome the primary resistance mechanism to ABT-263. Subsequent investigations validated this hypothesis. We demonstrated that the dual treatment of fatostatin and ABT-263 synergistically impaired the viability of various GBM cell types, including established cell lines, multiple patient-derived primary cultures and stem-like cells. Importantly, this synergistic reduction in cellular viability was confirmed in a more physiologically relevant three-dimensional (3D) spheroid model. The underlying mechanism for this enhanced efficacy of the dual treatment was further clarified through apoptosis and molecular analyses. The synergistic reduction in viability was, at least in part, due to a robust pro-apoptotic shift and was caspase-dependent. In addition, we observed that fatostatin monotherapy induced a dose-dependent downregulation of Mcl-1 protein expression in both an established and a primary cultured GBM cell line. This molecular event is, to our knowledge, a novel finding, and it provides a compelling explanation for the observed synergy: the inhibition of SREBP-1/2 by fatostatin leads to a reduction of Mcl-1 expression, sensitizing the cells to the additional blow delivered by ABT-263, which inhibits Bcl-xL and Bcl-2, resulting in a potent pro-apoptotic shift. This strategy-targeting Mcl-1 to overcome resistance to BH3 mimetics has been widely suggested as a viable therapeutic approach in GBM as well as various malignancies, including leukemia and lung cancer [28,29,30,31,32]. Our extracellular flux analyses provided further insight into the multi-faceted action of the combination therapy on cellular energy metabolism. We found that the dual treatment led to a pronounced suppression of OXPHOS, which persisted throughout the mitochondrial stress test, and a decrease in the glycolytic rate as well, with the exception of a possibly compensatory increase in ECAR levels at baseline. This suggests that the metabolic stress from SREBP-1/2 inhibition, combined with the direct mitochondrial perturbation from the BH3-mimetic, results in a comprehensive collapse of energy metabolism. Since high local invasiveness is another hallmark of GBM [33], another important finding of our study is the anti-migratory activity induced by the combined treatment. While ABT-263 previously showed limited impact on GBM cell migration [34], the dual treatment with fatostatin and ABT-263 significantly and more effectively impaired both random cell movement and directed cell migration, hinting at the crucial contribution of SREBP inhibition for this effect. Similarly, previous work has suggested that SREBP-1a activation induces a pro-migratory phenotype in GBM cells [35] and that fatostatin reduces migration of prostate cancer cells [36]. Collectively, our results provide robust in vitro proof-of-principle for the efficacy of combining SREBP-1/2 and Bcl-xL/Bcl-2 inhibition as a novel therapeutic approach for GBM. Besides, a critical observation for clinical validity is that the combination treatment showed low toxicity to non-pathological human endothelial cells compared to its high cytotoxicity in GBM cells. Despite these promising findings, our research is subject to several limitations. First, while we utilized diverse cell lines, including patient-derived primary and stem-like cells, the results remain confined to in vitro and 3D spheroid models. Second, both ABT-263 and fatostatin face known challenges regarding Blood–Brain Barrier (BBB) penetration, which is a central hurdle for central nervous system drug development. While ABT-263 has progressed to clinical trials [12,13] and fatostatin has been tested in various in vitro and in vivo cancer models [7,8], overcoming the BBB remains critical. Encouragingly, recent work has already suggested novel methods, such as nanostructured lipid carriers, to improve the brain availability of SREBP inhibitors like fatostatin [37]. Similar delivery approaches have been explored for ABT-263 penetration [32]. Finally, future investigations are warranted to verify and precisely delineate the mechanistic link between SREBP-1/2 inhibition by fatostatin and the subsequent downregulation of Mcl-1. Understanding whether this regulation occurs at the transcriptional, translational, or post-translational level is essential for optimizing this combination strategy. Furthermore, given the nature of interfering with two key metabolic/survival pathways, continued rigorous examination of the toxicity profile in non-pathological cells is warranted. To the best of our knowledge, this study represents the first to propose and mechanistically support the combined therapeutic application of fatostatin and ABT-263 for GBM.

## 5. Conclusions

The dual inhibition of lipid metabolism via SREBP-1/2 using fatostatin and mitochondrial apoptosis via Bcl-xL/Bcl-2 using ABT-263 provides a promising “double-hit” strategy against GBM. The efficacy of this combination therapy could be in part explained by the downregulation of Mcl-1 induced by fatostatin. These findings support the development of multi-targeted metabolic and apoptotic therapies to improve the management of this devastating malignancy.

## Data Availability

As part of the present study, the publicly available “Tumor Glioblastoma-TCGA-540-MAS5.0-u133a” dataset was analyzed using the R2 Genomics Analysis and Visualization Platform (Academic Medical Center, University of Amsterdam, The Netherlands, http://r2.amc.nl). The data supporting the results reported in the article are deposited at the translational brain tumor research laboratory, Department of Neurological Surgery, Ulm University Medical Center, Albert-Einstein-Allee 23, D-89081 Ulm, Germany.

## Supplementary Materials

The following supporting information can be downloaded at https://www.mdpi.com/article/10.3390/pharmaceutics18101327/s1, Figure S1: Synergism of action of fatostatin and ABT-263 combined; (a) the effect of the dual treatment was assessed after performing MTT assays on U251, primary-cultured GBM cells and stem-like glioma cells as outlined in Figure 3. Fraction affected (Fa)–combination index (CI) diagrams were then generated using the CompuSyn software. Different combinations of substance concentrations are represented by different data points. Data points under the horizontal line (CI < 1) represent synergism of action, those on the line (CI = 1) additivity, while data above the horizontal line (CI > 1) indicate antagonism. Data are representative of three independent experiments; (b,c) column graphs of cleaved caspase 9 and cleaved caspase 3 expression of U251 and ULM-GBM-PC38 cells after treatment with either fatostatin, ABT-263 or both agents combined, for 6 h (b) or 24 h (c). Normalization to β-actin was applied. Columns, mean; bars, SD; n = 3.; Figure S2: Fatostatin leads to a dose-dependent reduction of Mcl-1 expression, while treatment with fatostatin and ABT-263 combined induces a downregulation of both Mcl-1 and Bcl-xL. U251 and ULM-GBM-PC38 cells were treated as outlined. Subsequently, whole cell extracts were collected and underwent Western blot analysis for Mcl-1, Bcl-xL and Bcl-2 expression. Normalization to β-actin was applied. Densitometric analysis was performed using Bio-1D^®^ (Vilber, Eberhardzell, Germany). Columns, mean; bars, SD; n = 3; (a) Mcl-1, Bcl-xL and Bcl-2 expression of cells subjected to fatostatin monotherapy in different concentrations, as described; (b,c) Mcl-1, Bcl-xL and Bcl-2 expression of cells treated using either fatostatin, ABT-263 or both substances combined, as indicated, for 6 h (b) or 24 h (c).

## Abbreviations

The following abbreviations are used in this manuscript:

GBM	Glioblastoma, IDH-wildtype
TMZ	Temozolomide
SREBP-1	Sterol Regulatory Element-Binding Protein 1
SREBP-2	Sterol Regulatory Element-Binding Protein 2
SCAP	SREBP cleavage-activating protein
Mcl-1	Myeloid cell leukemia 1
Bcl-xL	B-cell lymphoma-extra large
Bcl-2	B-cell lymphoma 2
ABT-263	Navitoclax
BBB	Blood–brain barrier
TCGA	The Cancer Genome Atlas
SREBF-1	Sterol Regulatory Element Binding Transcription Factor 1
SREBF-2	Sterol Regulatory Element Binding Transcription Factor 2
GSCs	Glioblastoma stem-like cells
MTT	3-(4,5-dimethylthiazol-2-yl)-2,5-diphenyltetrazolium bromide
DMSO	Dimethyl sulfoxide
PI	Propidium iodide
BH3	Bcl-2 homology domain 3
