# Supplementary material for: BH3 Profiling Identifies Apoptotic Priming by SREBP-1/-2 Inhibition and Synergistic Anti-Glioblastoma Activity When Combined with ABT-263 In Vitro
Source: Pharmaceutics. 2026 Oct 20;18(10):1327. doi: 10.3390/pharmaceutics18101327 (PMC13708444; doi:10.3390/pharmaceutics18101327)
Supplement: Supplementary file 1 [file pharmaceutics-18-01327-s001.zip › Figure S1.pdf]

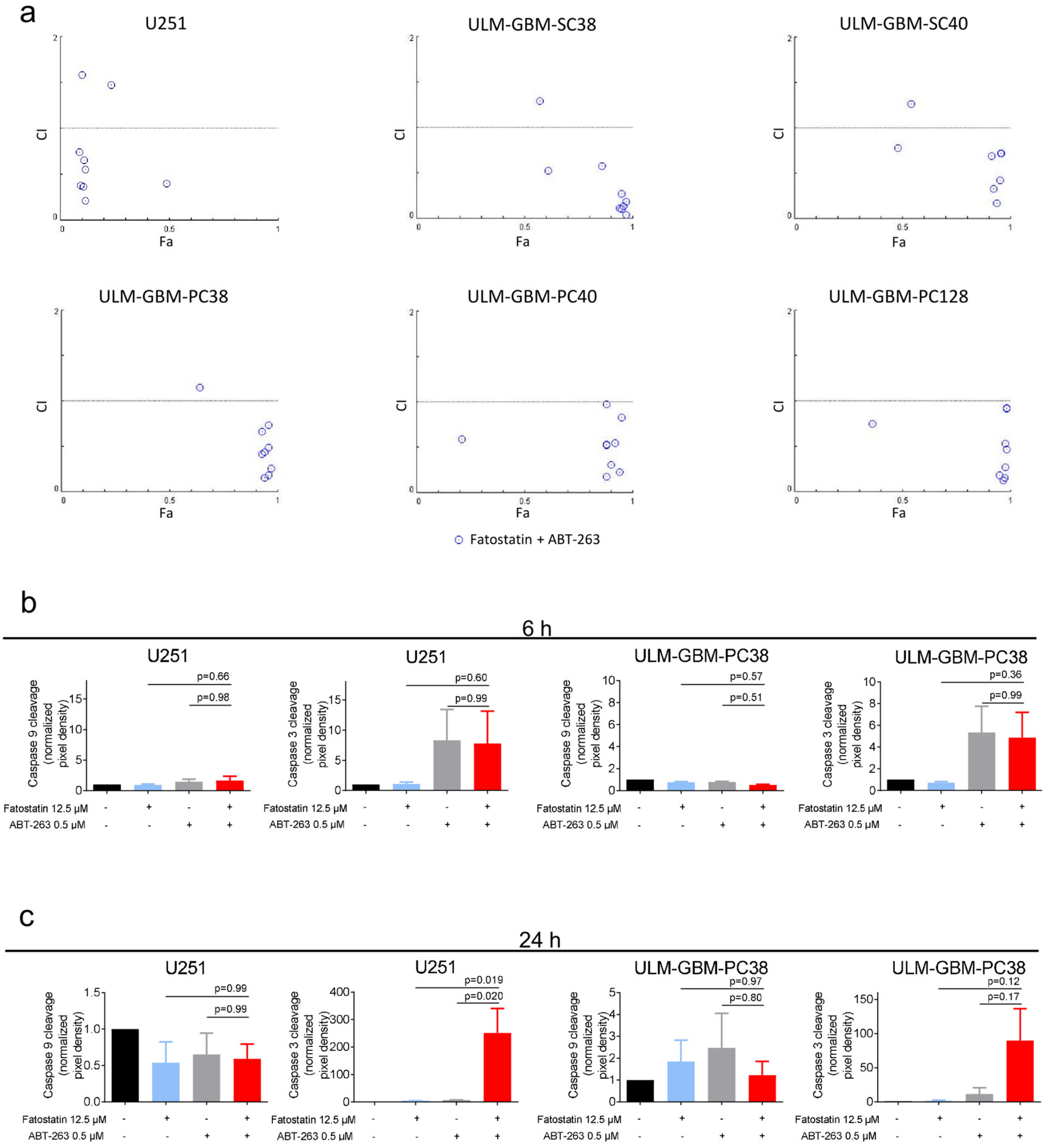

Supplemental Figure S1. Synergism of action of fatostatin and ABT-263 combined; (a) the effect of the dual treatment was assessed after performing MTT assays on U251, primary-cultured GBM cells and stem-like glioma cells as outlined in figure 3. Fraction affected (Fa) – combination index (CI) diagrams were then generated using the CompuSyn software. Different combinations of substance concentrations are represented by different data points. Data points under the horizontal line (CI < 1) represent synergism of action, those on the line (CI = 1) additivity, while data above the horizontal line (CI > 1) indicate antagonism. Data are representative of three independent experiments; (b, c) column graphs of cleaved caspase 9 and cleaved caspase 3 expression of U251 and ULM-GBM-PC38 cells after treatment with either fatostatin, ABT-263 or both agents combined, for 6 h (b) or 24 h (c). Normalization to β-actin was applied. Columns, mean; bars, SD; n = 3.
