# Supplementary material for: BH3 Profiling Identifies Apoptotic Priming by SREBP-1/-2 Inhibition and Synergistic Anti-Glioblastoma Activity When Combined with ABT-263 In Vitro
Source: Pharmaceutics. 2026 Oct 20;18(10):1327. doi: 10.3390/pharmaceutics18101327 (PMC13708444; doi:10.3390/pharmaceutics18101327)
Supplement: Supplementary file 1 [file pharmaceutics-18-01327-s001.zip › Figure S2.pdf]

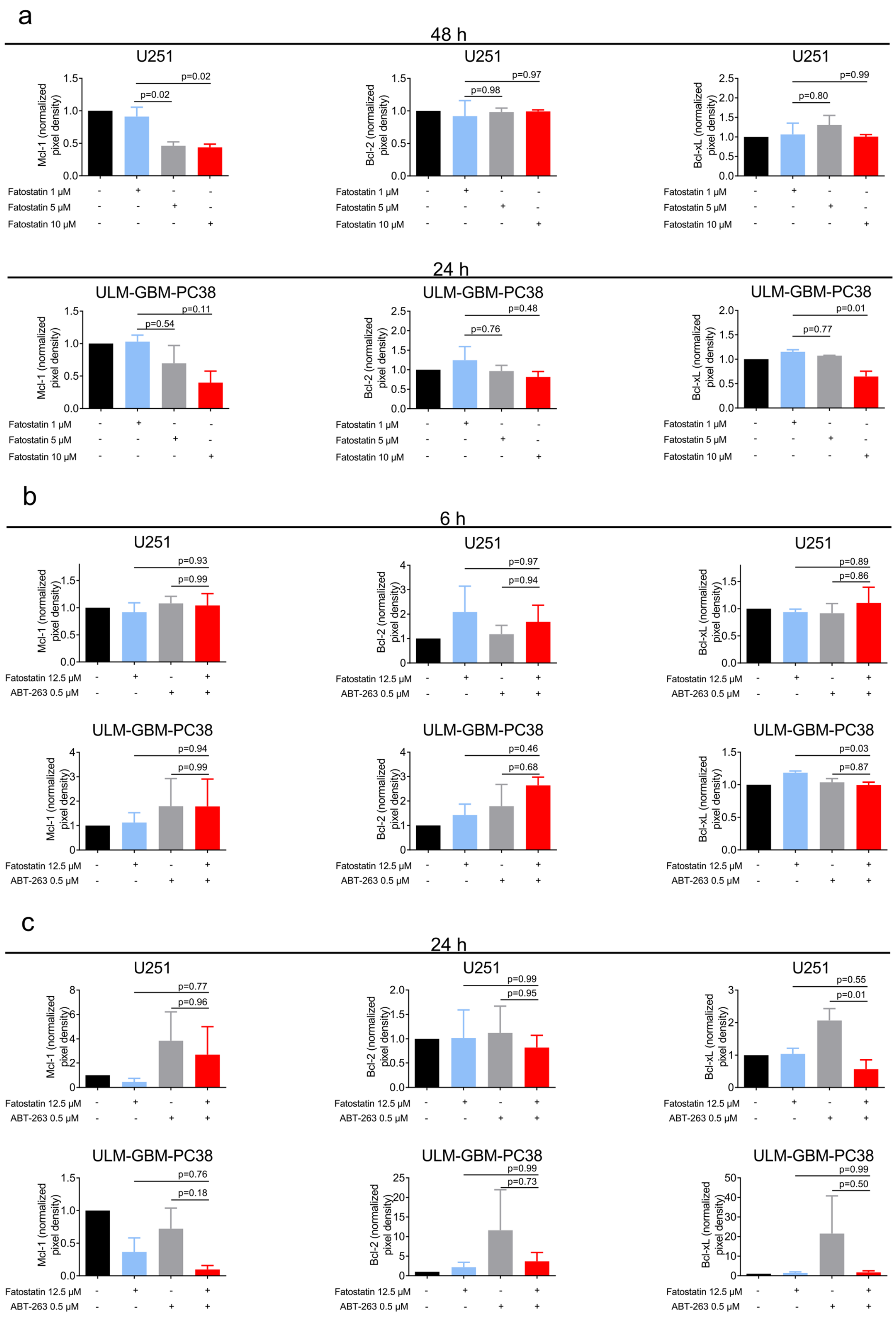
a
48 h
U251
Mcl-1 (normalized pixel density)
p=0.02
p=0.02
Bcl-2 (normalized pixel density)
p=0.97
p=0.98
Bcl-xL (normalized pixel density)
p=0.99
p=0.80
Fatostatin 1 µM
Fatostatin 5 µM
Fatostatin 10 µM
24 h
ULM-GBM-PC38
p=0.11
p=0.54
p=0.48
p=0.76
p=0.01
p=0.77
b
6 h
U251
p=0.93
p=0.99
p=0.97
p=0.94
p=0.89
p=0.86
Fatostatin 12.5 µM
ABT-263 0.5 µM
ULM-GBM-PC38
p=0.94
p=0.99
p=0.46
p=0.68
p=0.03
p=0.87
c
24 h
U251
p=0.77
p=0.96
p=0.99
p=0.95
p=0.55
p=0.01
ULM-GBM-PC38
p=0.76
p=0.18
p=0.99
p=0.73
p=0.99
p=0.50

**Supplemental Figure S2.** Fatostatin leads to a dose-dependent reduction of Mcl-1 expression, while treatment with fatostatin and ABT-263 combined induces a downregulation of both Mcl-1 and Bcl-xL. U251 and ULM-GBM-PC38 cells were treated as outlined. Subsequently, whole cell extracts were collected and underwent Western blot analysis for Mcl-1, Bcl-xL and Bcl-2 expression. Normalization to β-actin was applied. Densitometric analysis was performed using Bio-1D® (Vilber, Eberhardzell, Germany). Columns, mean; bars, SD; n = 3; (a) Mcl-1, Bcl-xL and Bcl-2 expression of cells subjected to fatostatin monotherapy in different concentrations, as described; (b, c) Mcl-1, Bcl-xL and Bcl-2 expression of cells treated using either fatostatin, ABT-263 or both substances combined, as indicated, for 6 h (b) or 24 h (c).
